# Impaction of permanent mandibular second molar: A retrospective study

**DOI:** 10.4317/medoral.18869

**Published:** 2013-03-25

**Authors:** Michele Cassetta, Federica Altieri, Alfonso Di Mambro, Gabriella Galluccio, Ersilia Barbato

**Affiliations:** 1 DDS,PhD Assistant Professor, Department of Oral and Maxillofacial Sciences, “Sapienza” University of Rome , Italy , School of Dentistry; 2 DDS Student,Department of Oral and Maxillofacial Sciences, “Sapienza” University of Rome , Italy , School of Dentistry; 3 Research Assistant, Department of Oral and Maxillofacial Sciences, “Sapienza” University of Rome , Italy , School of Dentistry; 4 Associate Professor, Department of Oral and Maxillofacial Sciences, “Sapienza” University of Rome , Italy , School of Dentistry; 5 Professor, Department of Oral and Maxillofacial Sciences, “Sapienza” University of Rome , Italy , School of Dentistry

## Abstract

Objective: To determine the prevalence of impacted mandibular second molar (MM2) and the association between MM2 impaction and crowding. The clinical significance of the angle between first and second mandibular molar and of the space between the first mandibular molar (MM1) and the anterior margin of mandibular ramus in MM2 impaction were also evaluated. Material and Methods: In this retrospective study , from the dental records of 2,945 caucasian young orthodontics patients, 40 subjects with MM2 impaction were included in a study group (SG) and compared with a control group (CG) of 200 subjects without MM2 impactions. The crowding, the angle of inclination of MM2, the distance between MM1 and mandibular ramus, the canine and molar relationships, and the lower centre line discrepancy were measured. For the statistical analysis , descriptive statistics and t-Student for independent sample groups were used. Results: The prevalence of impacted MM2 was 1.36%. The independent-Samples t-Test between SG and CG showed: the presence of crowding (P≤0.001), an higher angle values of MM2 inclination (P≤0.001) and a smaller distance between MM1 and the anterior margin of mandibular ramus (P≤0.001) in the SG. Conclusion: The impaction of MM2 is a relatively rare occurrence in orthodontic caucasian populations. The crowding, a higher angle values of MM2 inclination and a reduced distance between MM1 and the anterior margin of mandibular ramus, at the time of one third of MM2 root formation (T1), characterize MM2 impaction.

** Key words:**Impacted mandibular second molar, impaction, orthodontics.

## Introduction

Impaction of mandibular second molar (MM2) is relatively rare, with a reported prevalence of 0% - 2.3% (1-9), but it has been observed an increase in prevalence over the years (4,8,10,11). MM2 impaction has been described in three forms of angulations: mesial, vertical or distal (5). Mesial angulation is the most common form (5) and it appears that an initial inclination of MM2 greater than 20°/24° (4,12) or 30° (13) was associated with an higher impaction risk (4,5,12,14).

The aims of the current study were to determine:

- The prevalence of impacted MM2 in orthodontic caucasian young subjects.

- The association between MM2 impaction and crowding.

- The clinical significance of the angle between first and second mandibular molar in MM2 impaction.

- The clinical significance of a reduced back space between the distal height of the contour of the MM1 and the anterior margin of mandibular ramus in MM2 impaction.

The hypotheses of this study were that the MM2 impaction is relatively rare in caucasian populations and is associated with crowding. The authors also hypothesized that an increase in the angle between first mandibular molar and MM2 and a reduced back space between the distal height of the contour of the MM1 and the anterior margin of mandibular ramus , at the time of one third of MM2 root formation (T1), are further associated with MM2 impaction.

## Material and Methods

In this retrospective study, carried out at the Department of Orthodontics of “Sapienza” University of Rome from July 2011 to July 2012, a MM2 was considered impacted when it remains unerupted beyond the time when it should normally erupt, or when it is obvious that it is not going to erupt spontaneously (5). The impaction diagnosis and impaction site were determined on the basis of clinical examinations and panoramic radiographs. From dental records of 2,945 caucasian young orthodontics patients examined within one year, 40 subjects with MM2 impaction were included in a study group (SG) and compared with a control group (CG) of 200 caucasian subjects, without MM2 impactions, randomly selected from 1,667 remaining patients that satisfied the following criteria:

- caucasian children older than 10 years.

- The availability of two panoramic radiographs with a magnification rate of 1:1. The first at the time of one third of MM2 root formation (T1), and the second at the time of two thirds of MM2 root formation (T2).

- Good-quality of radiograms.

- Study models in T1.

All subjects with MM2 impactions also fulfilled the above criteria.

**Figure 1:**
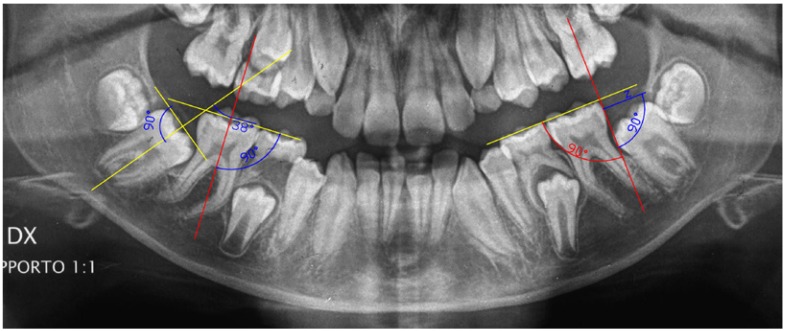
Panoramic radiograph , with a magnification rate of 1:1 , at the time of one third of MM2 root formation (T1): The measurements of the angle of inclination of MM2 ( right side ) and of the distance from the distal height of the contour of the first mandibular molar (MM1) to the anterior margin of mandibular ramus (left side) are shown.

On the panoramic radiographs were drawn linear and angular measurements:

Angle of inclination of MM2: measured, as described by Evans (4), in T1 and T2 (Fig. 1).

- Distance from the distal height of the contour of the first mandibular molar (MM1) to the anterior margin of mandibular ramus: a line perpendicular to the line passing through the occlusal or biting surfaces of the teeth and passing through the distal edge of the first molar was drawn. Then it was drawn a line parallel to the occlusal plane (line z) and tangent to the line passing through the distal edge of the cusp. The length of this line z, until reaching the anterior margin of the mandible, is the distance measured (Fig. 1).

- The distance was measured only in T1.

Study models allowed to measure:

- Crowding: Arch length analysis was a measurement of the discrepancy between space available (arch perimeter) and total tooth size (the sum of the mesio-distal widths of all the teeth within the arch) in T1. The degree of crowding within the arch is determined by subtracting the space available from the space required and the result is expressed directly in millimeters. For subjects in mixed dentition the Tanaka-Johnston’s prediction formula has been used.

- Canine and molar relationships: the Angle classification (class I-II-III) was used in T1.

- Lower centre line discrepancy measured in T1.

- All measurements were done twice by a single examiner and the average of two measurements recorded.

The local ethical committee was informed about the study protocol. We have read the Helsinki Declaration and followed the guidelines in the present investigation.

Statistical Analysis 

The reproducibility of the method was assessed by reexamining randomly 20 subjects 2 weeks after the first examination. Reproducibility was 100% for all variables except for crowding evaluation (97%) and angle of inclination of MM2 (98%). Statistical descriptive analysis was performed and the data were analyzed using SPSS software (Statistical Package for the Social Sciences, IBM Corporation, New York, NY). The statistical analysis was conducted at level of teeth. The statistical analysis was conducted at level of patients only in the assessment of the prevalence of MM2 impaction and of the distribution of subjects according to age and sex. Descriptive statistics consisting of mean, minimum-maximum and standard deviation were calculated for each group. Considering only the crowding, the angle of inclination of MM2 and the distance from the distal height of the contour of the MM1 to the anterior margin of mandibular ramus, the differences between SG and CG were determined. The differences in the mean were assessed by t-Student for independent sample groups. Results were assumed to be significant when the P value was ≤ 0.001.

## Results

Dental records of 2,945 subjects (1,567 females and 1,378 males; sex ratio: 4:3) were examined. From the study sample, 40 subjects were found with 57 impacted MM2 (23 male and 17 female; sex ratio 4:3 ). The prevalence of impacted MM2 was 1.36% (IC:95%).

Mesio-angular impaction was the most frequent (87.7%), while few molars were in disto-angular(10.5%) and only one in vertical impaction (1.8%). The angle of mesially impacted teeth ranged from + 1° to + 85° (mean:+34.77°). The angle of distally impacted teeth ranged from -15° to -3° ( mean:-7.17°). Bilateral impaction was seen in 17 patients, corresponding to 42.5%. Among the 23 patients with unilateral impaction, 17 impactions were seen on the right side (42.5%) and 6 on the left side (15%). In only 9 patients on 23 with unilateral impaction there was a lower centre line discrepancy on the same side of impaction. Both in SG and CG, the Class II molar and canine malocclusion was the most frequent ( Table 1). Mandibular third molar was present in all SG subjects, whereas was present in 89.9% of CG subjects. An average mandibular crowding of -0.42mm was recorded in the SG. No crowding was recorded in the CG (+2.86mm).

**Table 1:**
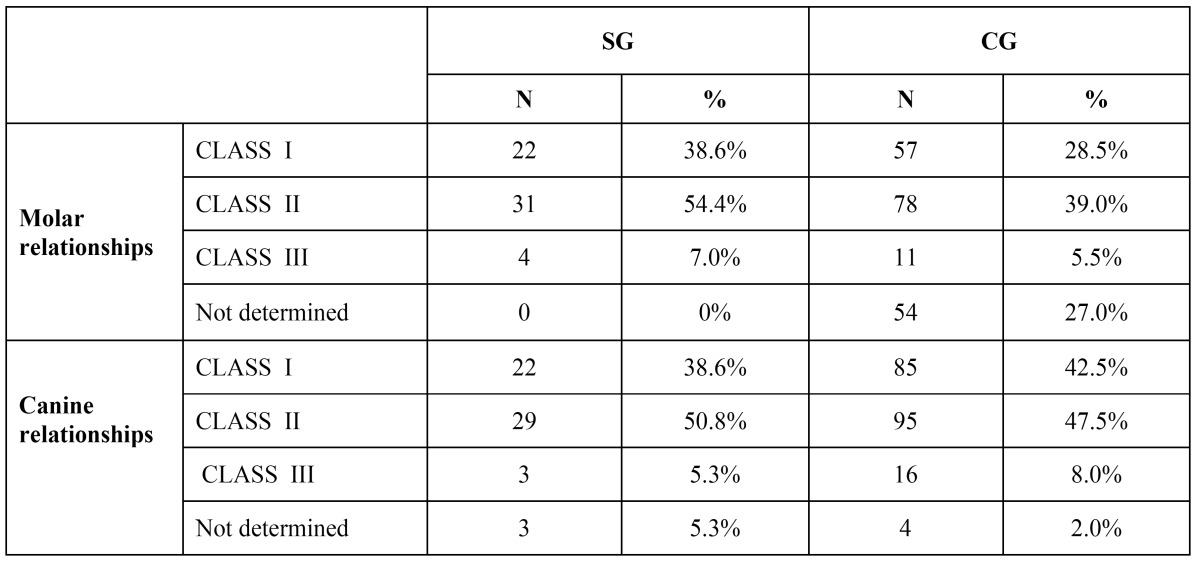
Occlusal relationships in the SG and CG following the Angle classification(classes I, II, III)( n=457).

Considering crowding, the paired comparison between SG and CG showed a statistically significant difference (P≤0.001) ( Table 2). The mean angle of inclination of MM2 in T1 was 29.12° for the SG, whereas 10.14° for GC, with a statistically significant difference (P≤0.001) ( Table 2). Considering the distance between MM1 and the anterior margin of mandibular ramus (line z) it was found smaller in the SG(mean: 9.20mm) than in the CG(mean: 12.8mm) with a statistically significant difference (P≤0.001) ( Table 2).

**Table 2:**
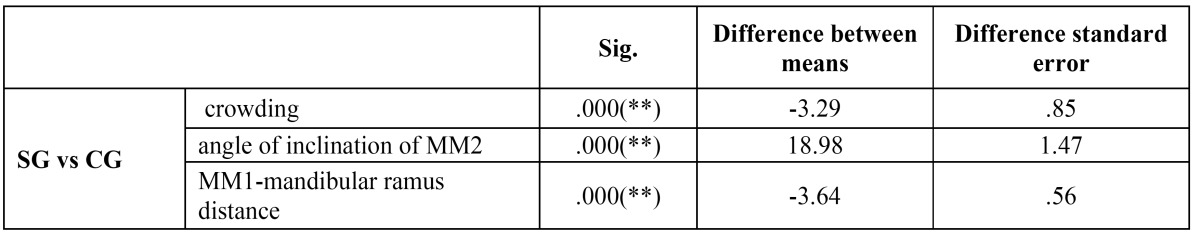
T-test between SG and CG evaluating crowding, angle of inclination of MM2, and MM1-mandibular ramus distance (P≤0.001)(n=457).(**) Statistically significative.

## Discussion

The prevalence of impacted MM2 in this study was relatively high (1.36%); this is probably due to an overestimation, because the study population was an orthodontic population. This outcome, however, reflects the increase in prevalence reported over the years by other studies (4,8,10,11,15). This incidence value is similar to that found by Evans (4) and suggests that the decreasing rate of extraction of first molars could be responsible for the increasing trend toward MM2 impaction.

Considering that the percentages of extraction cases in orthodontic treatment have shown a steady decline from 35% in 1986 to 18% in 2008, the increased frequency of impaction of the second mandibular molars may, likewise, arise from this different therapeutic approach (16). In the present study more males than females presented an impacted MM2 and the impaction was more common on the right side than on the left. This result agrees with Varpio and Wellfelt (5) that described more eruption disturbances of MM2 in males than females; bilateral impaction was seen in 23% of the cases, with a predominance of impaction on the right side (5). Differently, Cho et al. (11) showed a male-to-female ratio of 1:1.7 with a higher frequency of unilateral cases on the left side. Fu et al. (10) reported a male-to-female ratio of 1:1.12, with a higher frequency of unilateral impaction on the right side. Therefore, according to Fu et al. (10), no conclusion can be made as to whether any correlation exists between sex and impacted MM2. Previous studies have found that third molar adjacent to an impacted second molar is seldom missing (4-6,11).

Sonis and Ackerman (12) found no statistical significance between third molar presence and second molar impaction; Cho et al. (11) highlighted that mandibular third molars were developing in all but one case of MM2 impaction. Varpio and Wellfelt (5) also described the presence of third molar adjacent to the second molar in all but five of 88 patients with MM2 impaction. The present study confirmed the findings of the cited studies. Mandibular third molars were seen developing in the panoramic radiographs in all MM2 impaction cases. As mentioned by Vedtofte et al. (6), it is remarkable that all patients with MM2 impactions had the germ of the third permanent molar, which is normally only seen in 63.4-77.5 %. A relationship between the presence of third molar and MM2 impaction can be hypothesized. In this study, only 1 impacted MM2 showed a vertical position and 6 impacted MM2 showed a distal inclination; the remaining 50 MM2 were mesially impacted; the higher frequency of mesial inclination agrees with other previous authors (4,5,11).

Varpio and Wellfelt (5) referring to about 108 impacted MM2, showed a higher frequency of molars in mesioangular position forty-six and a lower frequency of molars in a distoangular and vertical position thirty-two and thirty, respectively . In the sample described by Cho et al. (11) only 1 on 42 MM2 impacted had a negative inclination; all other were mesially impacted. The angle of the mesially impacted teeth ranged from 13° to 75°. Evans (4) described an angle of mesially impacted teeth ranged from 15° to 65°. Fu et al. (10) pointed out that the angles of impacted MM2 were between 31° and 60°. In our study group, the angle range of mesially impacted teeth was similar to other study (5,11). One of the reasons for this skewed distribution, as affirmed by Raghoebar et al. (17), may be that during early development all mandibular molars are mesially inclined. In the present study a high percentage of Class II malocclusion, both in SG and CG, was found. This data is likely due to the study population that is represented by a selected orthodontic population where the Class II is the most frequent. In the current study we found a statistically significant association between inclusion of MM2 and crowding. Crowding has been cited as a common cause of MM2 impaction (4,5,11,13). In 1973, Buchner (18) stated that impaction of MM2 is a consequence of both anterior and posterior crowding. Evans (4), considering the possible MM2 impaction predisposing factors, stated that the most significant is the moderate to severe crowding. The same author (4) noted that unilateral impactions were associated with a center line discrepancy, with the lower center line shifted to the impacted side. Evans (4) indicated that this evidence is probably due to an asymmetry of crowding in the affected quadrant, resulting in a deficiency of space for full eruption of MM2. Varpio and Wellfelt (5) stated that the prevalence of impacted MM2 might thus be related to the orthodontic treatment philosophy, in fact, if expansion is preferred to extraction in cases of slight crowding, the prevalence of impacted MM2 will increase. The same authors (5) pointed out that among the three categories of angulation, impaction in mesioangular and in distoangular position seems mostly to have been caused by lack of space, while there appears to have been a number of local factors behind the vertical impaction (i.e. ankylosis, follicular cyst, supernumerary tooth, presence of fibromatose gingiva covering the impacted tooth). Magnusson and Kjellberg (9) reported an association between crowding and MM2 impaction in 70% of 166 second molars impacted. Differently, Shapira et al. (15) suggested that an excess of space in the molar region may lead to the impaction of MM2. In the present study, the distance between MM1 and the ramus was found smaller in the SG compared to CG with a statistically significant difference. Kaplan (19) suggested that MM2 impaction is usually a problem of arch length deficiency that may be associated with a third molar impaction.

Studies examining the impact of available space for mandibular third molar eruption have determined an association between available space and likelihood of impaction (20). Similarly to what occurs in case of the mandibular third molar impaction, although not explored as part of the present study, it is likely that any biomechanical approach that prevents mesialization of the first mandibular molar should determine a mandibular second molar impaction (12). According to this study results, a reduced back space in the mandibular molar area could be an etiopathogenetic local factor of MM2 impaction. As hypothesized by Kaplan (19) evaluating the factors related to mandibular third molar impaction, another important factor in determining MM2 impaction can be the insignificant resorption along the anterior border of the ramus.

## Conclusions

•The impaction of a mandibular second molar is a relatively rare occurrence with a prevalence in an orthodontic caucasian population, of 1.36%;

•The crowding, a higher angle of inclination of MM2, and a smaller distance between MM1 and mandibular ramus characterize the MM2 impaction.
